# Risk factors for pulmonary complications after hepatic resection: role of intraoperative hemodynamic instability and hepatic ischemia

**DOI:** 10.1186/s12871-017-0372-9

**Published:** 2017-06-20

**Authors:** Victoria Lepere, Antoine Vanier, Yann Loncar, Louis Lemoine, Jean Christophe Vaillant, Antoine Monsel, Eric Savier, Pierre Coriat, Daniel Eyraud

**Affiliations:** 1 Department of Anesthesiology and Reanimation, Assistance Publique-Hôpitaux de Paris (AP-HP), University Hospitals Pitié-Salpêtrière Charles-Foix, 43-87 Boulevard de l’Hôpital, 75013 Paris, France; 2 Department of Biostatistics, Sorbonne University, UPMC University, Paris 06, Paris, France; 3 Department of Biostatistics Public Health and Medical Informatics University Hospitals Pitié-Salpêtrière Charles-Foix, AP-HP, 43-87 Boulevard de l’Hôpital, 75013 Paris, France; 4 Department of Digestive, HPB Surgery, and Liver Transplantation University Hospitals Pitié-Salpêtrière Charles-Foix, AP-HP, 43-87 Boulevard de l’Hôpital, 75013 Paris, France

**Keywords:** Hepatectomy, Pulmonary complications, Hepatic ischemia, Vasopressors, Gamma-glutamyltransferase (GGT), Ischemia reperfusion, Lung injury

## Abstract

**Background:**

Postoperative operative pulmonary complications (PPCs) after hepatic surgery are associated with increased length of hospital stays. Intraoperative blood transfusion, extensive resection and different comorbidities have been identified. Other parameters, like time of hepatic ischemia, have neither been clinically studied, though experimental studies show that hepatic ischemia can provide lung injury. The objective of this study was to determinate the risk factors of postoperative pulmonary complications (PPCs) after hepatic resection within 7 postoperative days.

**Method:**

Ninety-four patients consecutively who underwent elective hepatectomy between January and December 2013. Demographic data, pathological variables, and preoperative, intraoperative, and postoperative variables had been prospectively collected in a data base. The dependant variables studied were the occurrence of PPCs, defined before analysis of the data.

**Results:**

PPCs occurred in 32 (34%) patients. A multivariate analysis allowed identifying the risk factors for PPCs. On multivariate analysis, preoperative gamma-glutamyltransferase (GGT) elevation OR =5,12 [1,85-15,69] *p* = 0,002, liver ischemia duration OR = 1,03 [1,01-1,06] *p* = 0,01 and the intraoperative use of vasopressor OR = 4,40 [1,58-13,36] *p* = 0,006 were independently associated with PPCs. For every 10 min added in ischemia duration, the OR of the risk of PPCs was estimated to be 1.37 (CI_95%_ = [1.08-1.81], *p* = 0.01).

**Conclusion:**

Three risk factors for PPCs have been identified in a population undergoing liver resection: preoperative GGT elevation, ischemia duration and the intraoperative use of vasopressor. PPCs after liver surgery could be related to lung injury induced by liver ischemia reperfusion and not solely by direct infectious process. That could explain why factors influencing directly or indirectly liver ischemia were independently associated with PPCs.

**Electronic supplementary material:**

The online version of this article (doi:10.1186/s12871-017-0372-9) contains supplementary material, which is available to authorized users.

## Background

Liver resection is an increasingly common surgery and remains the main metastatic colorectal disease and hepatocarcinoma treatment [1]. Despite technical advances and high experience of liver resection of specialized centres, it is still burdened by relatively high rates of postoperative morbidity and mortality [2]. Postoperative pulmonary complications (PPCs) were the most frequent complication in this series than in others, where the prevalence can reach up to 50% of the patients [3–7].

Many risk factors for PPCs have already been identified, such as advanced age, smoking status, history of chronic respiratory obstructive disease, ASA classification and incision site [8, 9]. These factors have been established for general elective surgery or upper abdominal procedures. Many fewer studies have specifically analysed the risk factor for PPCs after hepatectomy for cancer [4].

The clamping of the liver is a technique permitting decreasing intraoperative blood loss and thus perhaps decreasing the postoperative complications. However, this technique is followed by hepatic ischemia and then possibly lung injury by ischemia reperfusion syndrome. Although the role of liver and gut ischemia reperfusion has been well described for more than 20 years in animals [10, 11], few studies have specifically analyzed this phenomenon in humans. Thus, the aim of this study is to determinate the pre- and intraoperative risk factors of PPCs after hepatic resection, and especially to determine the role of hepatic ischemia, pre-operative biologic blood tests and the necessity of intraoperative vasopressive drugs in PPCs.

## Methods

### Design

Prospective cohort study of patients undergoing liver surgery. Risk factors for PPCs was studied.

### Setting

Study was performed in Department of Digestive, HPB Surgery, and Liver Transplantation and ICU at the Pitié-Salpêtrière Hospital Paris, France.

### Participants

All adult patients who were candidates for elective liver surgery between January 2013 and December 2013 in our centre were considered eligible for this study. There were no exclusion criteria. Data were prospectively collected in the data basis of our Hospital (MetaVision® Suite, Clinical Information System - version 5.47, Europe-iMDsoft GmbH, Germany).

#### Preoperative assessments

Each patient had a complete preoperative workup including a thoracoabdominopelvic computed tomographic (CT) scan with injection, a blood cell count, a coagulation profile with prothombin time, and a liver functional test [aspartate aminotransferase (AST), alanine aminotransferase (ALT), total bilirubin, alkaline phosphatase APL, gamma-glutamyltransferase (GGT)]. The future liver remnant after hepatic resection was calculated with helical three-dimensional CT scan (volumetry) [12].

### Anaesthetic, surgical procedure and perioperative care

#### Anaesthetic procedure

Each patient had right radial artery, and a right central venous catheter. Fluid management was left to the anaesthetists’ decision depending on the hemodynamic condition. The objective mean arterial pressure was >60 mmHg in the absence of comorbidity and >70 mmHg for patients with a cardiac risk. Blood transfusion was performed if the intraoperative haemoglobin level (measured with Hemocue) was <8-9 g/dl depending on the patients’ comorbidities. Precisely, hemoglobinemia of patients with cardiac disease was maintained >9 g/dl and more restrictive transfusion threshold <8 g/dL for the others. All patients received protective ventilation, defined by a tidal volume of 7-8 ml/kg, PEEP 5, FiO2 40-50%, recruiting manoeuvres were executed if needed.

### Surgical procedure

Liver resection was performed through abdominal incision or laparoscopy. Resection was performed without any clamping, with portal triad clamping (PTC) (intermittent or continuous) or total vascular exclusion of the liver (clamping of portal triad and infrahepatic and suprahepatic inferior vena cava). Total vascular exclusion of the liver (TVEL) was preceded by a test of few minutes of clamping to evaluate the hemodynamic tolerance and adapt the procedure [13].

### Postoperative management and data collection

All patients were admitted in the Intensive Care Unit during the early postoperative period. As soon as patients were stabilized, they were sent to the surgical ward.

Demographic data, comorbidity, pathological variables, operative and postoperative variables such as postoperative pulmonary complications were recorded. Biological parameters such as a liver functional test (AST, ALT, total bilirubin, alkaline phosphatase and gamma-glutamyltransferase), coagulation profile (PT, V factor), and blood cell counts were recorded on postoperative day (POD) 1, 3, 5 and 7. Routine chest radiography was performed on POD 1 and 3 and if pulmonary complication was suspected. In suspected cases of postoperative complications such as abdominal, pulmonary a thoracoabdominopelvic CT scan was performed. Patients were monitored for postoperative complications, mortality and the length of postoperative stay.

### Definitions

Postoperative pulmonary complications were defined as the development of one or more of the following conditions within 7 days of postoperative time: an Acute Respiratory Distress Syndrome (ARDS) defined as a ratio of Pa02/FiO2 < 200 [14], and bilateral opacities not explained by cardiac failure or fluid overload on chest radiology [1], pneumonia defined as temperature > 38,5°, purulent mucus and typical pulmonary imaging on chest radiology [15], drained pleural effusion, pulmonary embolism, patient still mechanically ventilated on POD 2, or needing oxygen therapy >3 L O2/min on POD2 (Capillary saturation in oxygen in ambient air <90%).

Cardiomyopathy was defined as Left Ventricular Ejection Fraction <40% at systematic preoperative echocardiography.

Echography was used in postoperative time to rule out cardiac failure in patients presenting ARDS.

Major hepatectomy was defined by the resection of three or more hepatic segments.

Chronic obstructive pulmonary disease (COPD) was diagnosed by spirometry [16, 17].

Hepatopathy was defined by the presence of steatosis >30% and/or suspicion of cirrhosis with preoperative imagery, confirmed with direct intraoperative analysis and histology.

Requirement of vasopressors was defined as a mean arterial pressure less than 65 mmHg for more than 5 min, despite adjusted volemia, and in absence of surgical venous obstruction.

Hepatic ischemia: portal triad clamping was performed, either continuous or with an alternate period of 10 min clamping and period of 5 min declamping. These patients (intermittent ischemia) were considered having hepatic ischemic time < 30 min.

### Statistical analysis

We used descriptive analyses with counts (and proportions), and means (with SDs), to describe the characteristics of the whole sample of patients.

Baseline characteristics (general characteristics, general comorbidities, hepatic comorbidities, etiology of surgery) and perioperative characteristics (surgery characteristics, anesthetic characteristics) were compared two-by-two, between patients with at least one pulmonary complication at POD 7 versus patients without any pulmonary complications, by means of univariate logistics regressions.

To investigate which characteristics could be independent factors associated with PPCs, a multivariate logistic regression model was fitted on the data. Nonetheless, the procedure of model selection had to be appropriate to consider the modest sample size and the presence of strongly intercorrelated characteristics (e.g. characteristics about liver function such as AST, ALT, GGT…). Therefore, first, if characteristics for which a *p*-value was estimated under 0.10 after univariate logistic regressions could all be considered for the multivariate logistic regression model, when different characteristics representing the same phenomenon were found to be associated after univariate analyses, only the one with the lowest *p*-value was considered for multivariate analysis (e.g. to represent liver function, only the characteristics that was the most associated after univariate analyses was included in multivariate analysis). Second, the procedure to select the best model was a stepwise (forward and backward) procedure, starting from a null model. The criterion that was used to select the best model was the Bayesian Information Criterion (BIC, the model with the lowest BIC was the one retained) which is a criterion designed to select a model based on the parsimony principle: only the factors that explained the best the data were retained relatively to the sample size.

Unadjusted Odds-Ratio (OR) after univariate analyses, and adjusted OR after multivariate analyses were estimated (with the absence of pulmonary complication as the reference), along with Confidence Interval at a 95% level (CI_95%_).

All statistical analyses were performed using R 3.0.2 [18].

## Results

Between January 2013 and December 2013, 94 consecutive patients underwent hepatectomy. In seven patients, laparoscopic surgery was performed: in four patients, minor hepatectomy and in 3 patients major hepatectomy. All patients presenting mean arterial pressure less than 65 mmHg for more than 5 min, despite adjusted volemia, and in absence of surgical venous obstruction received administration of Vasopressive drugs.

### General characteristics

Demographic and clinical characteristics of the model development sample are shown in Table 1.

**Table 1:** Patient characteristics, surgery characteristics and occurrence of pulmonary complications

Study sample (*n* = 94)	
Characteristic	Mean ± SD or n (%)
General characteristics	
Age	61 ± 13
Male gender	56 (60)
Body Mass Index	25 ± 5
Daily tobacco consumption	30 (32)
Daily alcohol consumption	13 (14)
ASA score > 2	50 (53)
General comorbidities	
COPD	9 (10)
Asthma	4 (4)
ASS	6 (6)
Cardiomyopathy	10 (11)
Chronic Kidney Disease	8 (9)
Diabetes	11 (12)
Hepatic comorbidities	
Non cancer hepatopathy	24 (26)
HCV infection	7 (7)
HBV infection	5 (5)
NASH	12 (12)
Alcoholic cirrhosis	4 (4)
Cirrhosis	19 (20)
Portal hypertension	8 (9)
Cholestasis	6 (6)
Liver underwent chimiotherapy	4 (4)
Etiology of surgery	
Primitive HCC	22 (23)
Metastasis	55 (59)
Primitive colic cancer	33 (35)
Primitive stomach cancer	4 (4)
Primitive breast cancer	4 (4)
Others	17 (18)
Surgery characteristics	
Major hepatectomy	45 (48)
Minor hepatectomy	40 (43)
Tumorectomy	9 (10)
Presence of clamping	69 (73)
by PTC	54 (57)
By TVEL	11 (12)
By PTC and TVEL	4 (4)
Intermittent	24 (26)
Continuous	45 (48)
Ischemia duration in min	26 ± 21
Bleeding volume in mL	529 ± 389
Duration of surgery in min	254 ± 89
Anesthetic characteristics	
Blood transfusion	27 (29)
blood volume transfused in mL	926 ± 562
Diuresis volume	398 ± 309
Use of vasopressive drug(s)	47 (50)
Pulmonary complications	
Presence of ≥1 complications (PPCs)	32 (34)
In those:	
ARDS	1 (1)
Pulmonary embolism	4 (4)
Drained pleural effusion	6 (6)
Pneumonia	13 (14)

*ASA score* American Society of Anesthesiology score, *COPD* chronic obstructive pulmonary disease, *SD* standard deviation, *ASS* apnoea sleep syndrome, *NASH* non alcoholic steatohepatitis, *HCC* hepatocellular carcinoma, *TVEL* Total vascular exclusion of the liver, *PTC* portal triad clamping, *ARDS* acute respiratory distress syndrome, *ASS* apnoea sleep syndrome, *PO* pulmonary oedema, *HCV* hepatitis C virus, *HBV* hepatitis B virus

#### Length of stay

The mean length of stay in ICU (intensive and light) was 4.3 ± 4 days.

Ten (11%) patients stayed in ICU, 88 (94%) in USI.

The mean length of stay in hospital was 6.1 ± 3.6 days.

#### Time of hepatic ischemia

Fourteen patients underwent TVEL, four of 15 with PTC + TVEL. The mean time of hepatic ischemia was 42 ± 11 min. Thirty patients underwent continuous PTC without TEVL with time of hepatic ischemia 36 ± 11 min. Twenty four patients underwent intermittent PTC with time of hepatic ischemia 39 ± 19 min. Twenty five patients underwent hepatic surgery without hepatic ischemia.

#### Mortality

The mortality was of 2%. Indeed, 2 patients died. One died of multiple organ failure at POD 20, he had ARDS which needed re-ventilation at POD 3 and ischemic colitis at POD 13. The second patient died of acute hepatic failure and multiple organ failure at POD 20. They both had cirrhosis and a continuous portal triad clamping of 45 min for the first patient and 30 min for the second.

### Factors associated with the presence of at least one PPCs (Main Analysis)

**Fig. 1:**
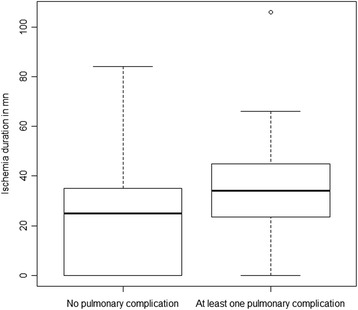
Distribution of ischemia duration by the presence or absence of PPCs

The univariate analysis allowed the identification of risk factors of occurrence of at least one PPCs (Table 2). This analysis identified quantitative factors such as the body mass index (BMI) (the mean BMI in PPCs vs No PPCs was of 26.3 ± 5.9 vs 24.0 ± 4.3, *p* = 0.04) and ischemia duration (34.3 ± 21.2 min in PPCs group vs 22.5 ± 20.5 min in No PPCs, *p* = 0.01 (Fig. 1)). This analysis identified qualitative factors such as the male gender (75% in PPCs group vs 52% in No PPCs group, *p* = 0.03), Daily alcohol consumption (28% in PPCs group vs 6% in No PPCs, *p* = 0.007), presence of cardiomyopathy (22% in PPCs group vs 5% in No PPCs, *p* = 0.02), the abnormality of GGT (71% in PPCs group vs 36% in No PPCs, *p* = 0.002), abnormality of AST (36% in PPCs group vs 57% in No PPCs, *p* = 0.07), ALT (26% in PPCs group vs 53% in No PPCs *p* = 0.01), APL, (7121% in PPCs group vs 48% in No PPCs *p* = 0.1), presence of clamping (66% in PPCs group vs 87% in No PPCs *p* = 0.01), clamping by TVEL (10% in PPCs group vs 28% in No PPCs *p* = 0.05) and the use of vasopressors (69% in PPCS group vs 40% in No PPCS group, *p* = 0.01). Table 2 displays the comparison of characteristics two-by-two, by the presence or absence of pulmonary complication.

**Table 2:** Comparison of characteristics two-by-two, by the presence or absence of pulmonary complication(s)

Variable and Level(s)	No PPC (*n* = 62)	PPC (*n* = 32)	p
	Mean ± SD or n(%)	Mean ± SD or n(%)	
General characteristics			
Age	60.9 ± 13.9	60.2 ± 12.9	0.81
Male gender	32 (52)	24 (75)	0.03
Body Mass Index	24.0 ± 4.3	26.3 ± 5.9	0.04
ASA score			0.42
1	3 (5)	1 (3)	
2	29 (47)	11 (34)	
3	30 (48)	20 (62)	
Daily tobacco consumption	17 (27)	13 (41)	0,20
Daily alcohol consumption	4 (6)	9 (28)	0,007
General comorbidities			
COPD	4 (6)	5 (16)	0,16
Asthma	3 (5)	1 (3)	0,70
ASS	3 (5)	3 (9)	0,40
Cardiomyopathy	3 (5)	7 (22)	0,02
Chronical kidney failure	7 (11)	1 (3)	0,21
Diabetes	5 (8)	6 (19)	0,14
Hepatic comorbidities			
Non cancer hepatopathy	11 (18)	13 (41)	0,02
Cirrhosis	9 (14)	10 (31)	0,06
Portal hypertension	4 (6)	4 (12)	0,33
NASH	6 (6)	6 (6)	0,33
Cholestasis	3 (5)	3 (9)	0,40
Liver underwent chemiotherapy	3 (5)	1 (3)	0,70
Pre-surgery biological workup			
Hemoglobin level in g/dL	12.9 ± 1.7	13.2 ± 1.9	0.44
Platelets level in G/L	227 ± 97	215 ± 68	0.55
White blood cells level in G/L	7.3 ± 6.1	7.1 ± 2.2	0.83
Prothrombin ratio	99.1 ± 12.4	98.1 ± 14.1	0.74
Fibrinogen level in g/L	4.1 ± 1.3	4.4 ± 1.4	0.35
Creatinin level in μmol/mL	86.2 ± 52.3	76.6 ± 19.6	0.36
Abnormal GGT	21 (36)	22 (71)	0,002
Abnormal AST	21 (36)	17 (57)	0,07
Abnormal ALT	15 (26)	16 (53)	0,01
Abnormal total Bilirubin	10 (17)	3 (11)	0,51
Abnormal APL	10 (21)	14 (48)	0,01
Etiology of surgery			0.17
Primituve HCC	11 (18)	11 (34)	
Metastasis	38 (61)	17 (53)	
Others	13 (21)	4 (12)	
Surgery charactetistics			
Major hepatectomy	23 (37)	22 (69)	0.005
Presence of clamping	41 (66)	28 (87)	0.10
Clamping by PTC	36 (58)	22 (69)	0,31
Clamping by TVEL	6 (10)	9 (28)	0,03
Ischemia duration in mn	22.5 ± 20.5	34.3 ± 21.2	0.01
Bleeding volume in mL	510.0 ± 417.3	562.9 ± 335.7	0.54
Duration of surgery in mn	246.2 ± 92.3	269.9 ± 80.0	0.22
Anaesthetic characteristics			
Blood transfusion	17 (27)	10 (31)	0,70
Diuresis volume	418.4 ± 337.4	362.8 ± 251.9	0.41
Use of vasopressive drug(s)	25 (40)	22 (69)	0,01

*PPC* postoperative pulmonary complication, *ASA score* American Society of Anesthesiology score, *COPD* chronic obstructive pulmonary disease, *SD* standard deviation, *ASS* apnoea sleep syndrome, *NASH* non alcoholic steatohepatitis, *HCC* hepatocellular carcinoma, *TVEL* Total vascular exclusion of the liver, *PTC* portal triad clamping, *ARDS* acute respiratory distress syndrome, *ASS* apnoea sleep syndrome, *PO* pulmonary oedema, *HCV* hepatitis C virus, *HBV* hepatitis B virus, *GGT* gamma-glutamyltransferase, *AST* aspartate aminotransferase, *ALT* alanine aminotransferase, total bilirubin, *APL* alkaline phosphatase

**Fig. 2:**
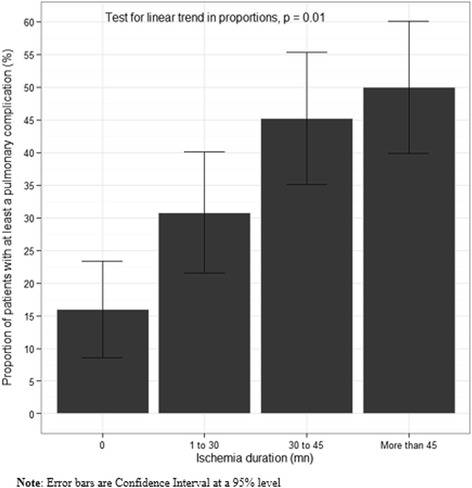
Rate of patients presenting PPC in relation with the time of hepatic ischemia

All factors associated with the presence of PPCs with a *p*-value <0.10 were considered for the multivariate analysis (Tables 2 and 3). After a model selection (see statistical analysis), the final multivariate analysis retained 3 risk factors independently associated with PPCs: preoperative elevated GGT (Adjusted OR = 5.12, CI_95%_ = [1.85-15,69], *p* = 0.002), ischemia duration (Adjusted OR = 1.03, CI_95%_ = [1.01-1.06], *p* = 0.01) and the use of vasopressor (Adjusted OR = 4.40, CI_95%_ = [1.58-13.36], *p* = 0.006) (Table 3). The proportion of patients with at least one PPC increased with ischemia duration: from 16.0% of patients with PPCs among surgeries with no hepatic ischemia to 50.0% of patients with PPCs among surgeries with hepatic ischemia duration of more than 45 min (test for linear trend in proportions *p* = 0.01, Fig. 2). For every 10 min added in ischemia duration, the OR of the risk of PPCs was estimated to be 1.37 (CI_95%_ = [1.08-1.81], *p* = 0.01).

**Table 3:** Unadjusted Odds-Ratio after univariate logistic regressions and adjusted Odds-Ratio after multivariate analysis

Variable	Univariate logistic regressions			Multivariate logistic regression		
	OR	CI_95%_	p	OR	CI_95%_	p
General characteristics						
Male gender	2.81	[1.13 – 7.57]	0.03	-	-	-
Quantitative Body Mass Index	1.10	[1.01 – 1.21]	0.04	-	-	-
Daily alcohol consumption	5.67	[1.67 – 22.67]	0.007	-	-	-
General comorbidities						
Cardiopathy	5.51	[1.41 – 27.18]	0.02	-	-	-
Hepatic comorbidities						
Non cancer hepatopathy	3.17	[1.22 – 8.45]	0.02	-	-	-
Cirrhosis	2.68	[0.96 – 7.64]	0.06	-	-	-
Pre-surgery biological workup						
Abnormal GGT	4.31	[1.72 – 11.50]	0.002	5.12	[1.85 – 15.69]	0.002
Abnormal AST	2.30	[0.94 – 5.76]	0.07	-	-	-
Abnormal ALT	3.28	[1.31 – 8.45]	0.01	-	-	-
Abnormal APL	3.55	[3.31 – 9.99]	0.01			
Surgery characteristics						
Major hepatectomy	3.73	[1.54 – 9.57]	0.004	-	-	-
Clamping by TVEL	3.65	[1.18 – 12.03]	0.03	-	-	-
Ischemia duration in min	1.03	[1.01 – 1.05]	0.01	1.03	[1.01 – 1.06]	0.01
Anaesthetic characteristics						
Use of vasopressive drug(s)	3.26	[1.35 – 8.31]	0.01	4.40	[1.58 – 13.36]	0.006

*GGT* gamma-glutamyltransferase, *AST* aspartate aminotransferase, *ALT* alanine aminotransferase, total bilirubin, *APL* alkaline phosphatase, *TVEL* total vascular exclusion of the liver

## Discussion

The ability to predict and treat early postoperative morbidity after elective liver resection would allow individualized postoperative management. In this prospective one-year study, we identified three independent risk factors for PCCs after hepatic resection: prolonged time of ischemia, the use of vasopressor and elevated preoperative GGT.

Firstly, it is shown that prolonged time of ischemia during a hepatectomy is a risk factor of PCC within 7 days of postoperative. In our study, the OR for time of ischemia corresponds to an increase in the risk with every extra minute of ischemia. Patients with PPC had undergone longer time of hepatic ischemia (Fig. 1) and the longer the time of hepatic ischemia the greater was the rate of PPC (Fig. 2). The mechanism explaining the link between hepatic ischemia and PPCs could be the massive release of reactive oxygen species factors and cytokine production. This process has been demonstrated in experimental studies with kidneys and extra renal organs such as the lung [19, 20]. Indeed, it has been shown that acute kidney injury leads to a pro inflammatory and pro apoptotic pathways activation which then leads to an inflammatory response in the lung, then to ARDS. This phenomenon has been also described for the liver in an experimental study: liver ischemia-reperfusion (I/R) increases pulmonary permeability in rats [10] and induced pathological changes in lung parenchyma (alveolar septal thickening, leukocyte infiltration, hemorrhage and pulmonary edema) in rabbits [21]. Because the lung function depends directly on the cell membrane integrity of the alveolar-capillary network of the lung, whose structure is particularly sensitive to factors related to I/R, as the decrease in cellular energy levels and the action of the reactive oxygen species, liver ischemia reperfusion can infer pulmonary edema and contribute to acute respiratory distress syndrome (ARDS).

Moreover, hepatic clamping results in venous inflow occlusion from the gut and therefore leads to gut ischemia reperfusion. Collange et al. [22] showed that gut ischemia increases lactate, cytokine IL10 IL6 in systemic blood. IL-10 was mainly found in the lung, suggesting the interaction between lung inflammation and gut ischemia-reperfusion. Karsten Bartels et al. [23] also show the effects of gut ischemia to the cellular scale. Moreover Ben-Abraham et al. [11] demonstrated acute lung injury induced by mesenteric artery clamping/unclamping. What’s more, experimental studies have demonstrated that a chemical medication could prevent lung injury in hepatic ischemia reperfusion: Uchiyama et al. [24] proved that Edavarone, a potent free-radical scavenge, could decrease lung injury in rats by attenuating oxidative stress response. This could maybe open the possibility of preventing PPCs after elective liver surgery.

Secondly, the use of vasopressor has been shown as a risk factor of PPC within 7 days of postoperative. The association between vasopressor and PPC could have two origins: first, the cause of administration of these drugs by the anaesthesiologist, hemodynamic instability, could be the cause of PPC: hemodynamic instability, whatever is its cause, could lead to inadequate lung perfusion in the intraoperative time and then to PPCs [25]. Second, vasopressor could have per se a deleterious effect. In a recent survey, authors [26] described the intraoperative fluid and pharmacologic management during liver transplantation in the USA. One of the conclusions was that the use of vasopressors was even less since the liver transplantation program was efficient.

In a recent large study analyzing pre- and intraoperative risk factors for ARDS after liver transplantation, authors showed that large intraoperative bolus of vasopressors were the sole intraoperative risk factor [27]. That points out the dilemma of the physician: such techniques as hepatic clamping or vasopressive drugs have a very interesting effect on the intraoperative blood loss and the quality of surgery it allows (with probably less postoperative surgical complications and a better treatment of the cancer). However hepatic clamping such as the use of vasopressors could have deleterious effect on the liver but also on other organs such as the lung. That should focus our efforts on patient-centered outcomes, which are clearly more important than process outcomes [28].

Thirdly, the third independent risk-factor for PPCs identified in our study was an elevated preoperative concentration of GGT. Because GGT and APL, are very sensitive parameters of liver diseases [29], it was rational to study if preoperative GGT could be predictive of PPCs. In a large cohort of 278,419 patients, recorded over 7 years, Sung KC et al. [30] have shown that GGT > 35 UI/l was an independent factor of mortality, not regarding the presence of fatty liver. In this Korean cohort, there was no significant association between ALT and all causes of mortality. Preoperative GGT has not often been studied in liver surgery. Rau HG et al. [31] analyzed the postoperative risk of liver failure in a series of 570 patients: preoperative GGT was one of the three independent parameters predicting liver failure. Because of the small number of patients with postoperative liver failure in our series, the preoperative GGT as a risk factor for liver failure could not be analysed. However, GGT and not transaminases, as for liver failure in the Rau’s study, was a risk factor for PPCs. The direct role of the liver in the association between elevation of GGT and postoperative morbidity could however be discussed. Indeed, some authors reported the association between baseline levels of GGT and specific cardiovascular complications [32–34] in the general population.

Also, the rate of PPC in our study was higher than other studies [4, 35, 36]. This can be explained by our large definition of pulmonary infection and the fact that we wanted specifically to study PPCs. Indeed, we defined pulmonary infection with large clinical criteria such as fever, expectoration of mucus and the presence of a suspect image on thoracic radiography. One criteria we used for PPCs is “oxygen therapy >3L O2/min on POD2”, that is not present clearly in Dindo-Clavien classification [37], could significantly increase the number of patients with PPC. In fact this criteria could be considered as Grade 2 complication of the Dindo-Clavien Classification, because, in our current practice most patient did not need oxygen therapy on POD 2. This was present in the criteria definition recommended by an ESA-ESICM statement [38].

It could be asked in what way the results of our study are dependent of the choice of the definition of PPCs. To explore this aspect, we have performed a sensitivity analysis where “oxygen therapy >3L O2/min on POD2” was not considered as a criterion for having at least one PPC. All the results can be found in Additional file 1. In short, according to this second definition, there would be 21 (22.3%) patients with PPCs instead of 32. Univariate comparisons by the presence or absence of PPCs would show results closed to the main analysis: four characteristics that were not found to be associated with PPCs would become associated (diabetes, portal hypertension, steatosis and etiology), and two characteristics that were associated to PPCs in the main analysis would become not associated (major hepatectomy and clamping by TVEL). All the others associations would remain the same. After performing multivariate logistic regression with the same procedure and criteria for model selection as the main analysis, the model that would be retained is a model with the same characteristics as the main analysis (GGT, ischemia duration and use of vasopressive drugs) alongside with two more characteristics (cardiopathy and non-cancer hepatopathy). To summarize, this sensitivity analysis confirms the three characteristics retained in the main analysis, but allow discussing the role of two other factors.

Finally, obesity, defined in our article by BMI > 30, was not demonstrated as a risk factor. This result could be compared with that of Cucchetti et al. [39]. COPD, like in the study of Nobili et al. [4] was not an independent risk factor and diabetes, in contrary of that study was also not an independent risk factor. The cause of this discrepancy could be the lack of power of our study, where the number of patients presenting COPD or diabetes was low. Another cause could be that the pathophysiology of PPCs after liver surgery was first liver ischemia reperfusion induced lung injury and not direct pulmonary infection. This hypothesis should be confirmed by a larger prospective study.

### Limitations of the study

The main limit of the study is the number of included patients and the fact it was a retrospective design (it is a study designed to generate hypotheses). Despite the modest sample size, three independent characteristics were found to be associated with PPCs, with a Type-1-error equal to 5%. As they were retained after a procedure of model selection starting from a null model and based on an information criterion (BIC), these 3 characteristics can be hypothesized as characteristics likely to be strongly associated with PPCs. Nonetheless, as sample size was modest, a lack of power is plausible. Therefore, it is plausible some other independent characteristics associated with PPCs were not retained in the final multivariate model due to lack of power. For example, it is impossible to disentangle if the presence of cardiomyopathy, a characteristic that was found to be associated with PPC in univariate analyses, is not in the final model because it is really not associated independently with PPC (we can hypothesize it is a cofounder: patients with cardiomyopathy have a higher probability to be patients for which a use of vasopressive drugs will be required during surgery, and the model has retained the use of vasopressive drug as the characteristic that matters), or because of a lack of power due to sample size. Bigger database is needed to include additional variables in the multivariate analysis to investigate the effects of confounders.

Another limitation is the definition of the vasopressive request, completely let to the evaluation of the anesthesiologist, however always senior anesthesiologist, specialized in liver surgery. A third limitation is the lack of inflammation biomarker to establish the relation of causality between ischemia-reperfusion, liver ischemia and pulmonary complications. At last, our choice to consider patients with intermittent ischemia having a priori hepatic ischemic time < 30 min, could be arbitrary. However our choice founded on our experience that intermittent clamping is safe, even in compromised livers, but should not be applied >120 min, and is not equivalent to the absence of liver ischemia. Moreover, most studies about liver ischemia corroborated our experience [40], even if authors were more interesting in hepatic tolerance than in extra hepatic complications.

### Practical applications

The main consequences of our study are to minimize liver ischemic time and to prefer intermittent ischemia, when possible, and to minimize the use of Vasopressive drugs. For this last point, two types of complementary monitoring are useful: first BIS, because episodes of hypotension could be related with too profound level of anaesthesia, and second hemodynamic optimization using arterial and central venous pressures or/and Stroke Volume Variation [41]: Vasopressive therapy is perhaps useful in some patients undergoing hepatic surgery but should be used only after control of adequate level of anaesthesia and volemia.

## Conclusions

PPC remains a frequent postoperative complication. Our study allowed identifying three independent risk factors of PPC: prolonged time of ischemia, the use of vasopressor and elevated preoperative GGT. Preoperative GGT is not controllable. However, use of vasopressors for correction of all episodes of arterial hypotension and the time of hepatic ischemia are completely decided upon by the medical team. The necessity of hepatic clamping is decided after surgical evaluation and the actual evolution is to minimize it or to prefer the intermittent. Concerning the treatment of intraoperative hypotension, definition of hypotension requiring vasopressors and the benefit/risk of such treatment should be probably finely defined in a randomized trial.

## Additional file

Additional file 1: Supplementary analyses. (DOC 120 kb)

## Abbreviations

ALT: Alanine aminotransferase; total bilirubin
APL: Alkaline phosphatase
ARDS: Acute respiratory distress syndrome
ASA score: American Society of Anesthesiology score
ASS: Apnoea sleep syndrome
AST: Aspartate aminotransferase
GGT: Gamma-glutamyltransferase
HBV: Hepatitis B virus
HCC: Hepatocellular carcinoma
HCV: Hepatitis C virus
ICU: Intensive care unit
NASH: Non alcoholic steatohepatitis
PO: Pulmonary oedema
POD: Postoperative day
PPCs: Postoperative operative pulmonary complications
PTC: Portal triad clamping
TVEL: Total vascular exclusion of the liver
