# Supplementary material for: Risk factors for pulmonary complications after hepatic resection: role of intraoperative hemodynamic instability and hepatic ischemia
Source: BMC Anesthesiol. 2017 Jun 20;17:84. doi: 10.1186/s12871-017-0372-9 (PMC5477742; doi:10.1186/s12871-017-0372-9)
Supplement: Additional file 1: — Supplementary analyses. (DOC 120 kb) [file 12871_2017_372_MOESM1_ESM.doc]

**Risk factors for pulmonary complications after hepatic resection. Role of intraoperative hemodynamic instability and hepatic ischemia**

# SUPPLEMENTARY ANALYSES

- **Analyses if the need for oxygen therapy >3L O2/min on POD2 (Capillary saturation in oxygen in ambient air < 90%) is not included in the definition of a presence of at least one pulmonary complication**

Therefore: Number of subjects with at least one pulmonary complication (PPC):

Yes = 21 (22.3%), No = 73 (77.7%)

SupTable1. Comparison of characteristics two-by-two, by the presence or absence of pulmonary complication(s) (second definition)

| **Variable and Level(s)** | **No PPC**  **Mean ± SD**  **or n(%)** | **PPC(s)**  **Mean ± SD**  **or n(%)** | **p** | **Variable and Level(s)** | **No PPC**  **Mean ± SD**  **or n(%)** | **PPC(s)**  **Mean ± SD**  **or n(%)** | **p** |
| --- | --- | --- | --- | --- | --- | --- | --- |
| *General characteristics* | | | | Prothrombin ratio | 99.1 ± 12.0 | 97.6 ± 16.1 | 0.66 |
| Age | 60.8 ± 13.4 | 60.3 ±14.3 | 0.98 | Fibrinogen level in g/L | 4.5 ± 1.4 | 4.0 ± 1.3 | 0.72 |
| Male Gender | 40 (55) | 16 (76) | 0.08 | Creatinin level in μmol/mL | 83.9 ± 49.6 | 80.2 ± 18.9 | 0.75 |
| Body Mass Index | 24.2 ± 4.4 | 26.7 ± 6.3 | 0.04 | Abnormal GGT | 28 (41) | 15 (75) | 0.01 |
| ASA |  |  | 0.18 | Abnormal ASAT | 25 (36) | 13 (68) | 0.02 |
| 1 | 4 (5) | 0 (0) |  | Abnormal ALAT | 20 (29) | 11 (58) | 0.02 |
| 2 | 33 (45) | 7 (33) |  | Abnormal total Bilirubin | 11 (16) | 2 (12) | 0.71 |
| 3 | 36 (49) | 14 (67) |  | Abnormal APL | 14 (25) | 10 (50) | 0.04 |
| Daily tobacco consumption | 21 (29) | 9 (43) | 0.22 | *Etiology of surgery* |  |  | 0.02 |
| Daily alcohol consumption | 6 (8) | 7 (33) | 0.006 | Primituve CHC | 12 (16) | 10 (48) |  |
| *General comorbidities* | | | | Metastasis | 47 (64) | 8 (38) |  |
| COPD | 5 (7) | 4 (19) | 0.11 | Others | 14 (19) | 3 (14) |  |
| Asthma | 3 (4) | 1 (5) | 0.90 | *Surgery charactetistics* | | | |
| OSAS | 4 (5) | 2 (9) | 0.51 | Major hepatectomy | 32 (44) | 13 (62) | 0.15 |
| Cardiopathy | 3 (4) | 7 (33) | 0.001 | Presence of clamping | 51 (70) | 18 (76) | 0.16 |
| Chronical kidney failure | 7 (10) | 1 (5) | 0.49 | Clamping by PTC | 43 (59) | 15 (71) | 0.30 |
| Diabetes melitus | 6 (8) | 5 (24) | 0.06 | Clamping by TVEL | 10 (14) | 5 (24) | 0.27 |
| *Hepatic comorbidities* | | | | Ischemia duration in mn | 23.9 ± 20.1 | 35.4 ± 23.7 | 0.03 |
| Non cancer hepatopathy | 12 (16) | 12 (57) | < 0.001 | Bleeding volume in mL | 511.5 ± 395.3 | 583.9 ± 371.3 | 0.46 |
| Cirrhosis | 10 (14) | 9 (43) | 0.005 | Duration of surgery in mn | 247.9 ± 86.9 | 276.5 ± 92.8 | 0.19 |
| Portal hypertension | 4 (5) | 4 (19) | 0.06 | *Anesthetic characteristics* | | | |
| Steatosis | 4 (5) | 4 (19) | 0.06 | Blood transfusion | 18 (25) | 9 (43) | 0.11 |
| Cholestasis | 5 (7) | 1 (5) | 0.73 | Protective ventilation | 58 (79) | 16 (76) | 0.75 |
| Liver underwent chemotherapy | 3 (4) | 1 (5) | 0.90 | Diuresis volume | 408.5 ± 317.3 | 365.7 ± 285.6 | 0.58 |
| *Pre-surgery biological workup* | | | | Use of vasopressive drug(s) | 32 (44) | 15 (71) | 0.03 |
| Hemoglobin level in g/dL | 13.0 ± 1.6 | 13.1 ± 2.2 | 0.93 | Hemodynamic instability | 6 (8) | 2 (9) | 0.86 |
| Platelets level in G/L | 230 ± 93 | 195 ± 60 | 0.15 |  |  |  |  |
| White blood cells level in G/L | 7.3 ± 5.6 | 7.0 ± 2.3 | 0.79 |  |  |  |  |

Note: Variables in green are variables that were not identified as associated with the presence of pulmonary complication according to the definition of the main analysis, but become associated when applying the definition described in page 1 of this supplementary material. Variables in red are variables that were found to be associated with the presence of pulmonary complication according to the definition of the main analysis, but were not found to be associated when applying the definition described in page 1.

SupTable2. Unadjusted Odds-Ratio after univariate logistic regressions and adjusted Odds-Ratio after multivariate analysis (second definition)

| **Variable** | **Univariate logistic regressions** | | | **Multivariate logistic regressions** | | |
| --- | --- | --- | --- | --- | --- | --- |
|  | **OR** | **CI_95%_** | **p** | **OR** | **CI_95%_** | **p** |
| *General characteristics* | | | | | | |
| Male gender | 2.64 | [0.92 – 8.76] | 0.08 | - | - | - |
| Quantitative Body Mass Index | 1.10 | [1.01 – 1.22] | 0.04 | - | - | - |
| Daily alcohol consumption | 5.58 | [1.63 – 19.99] | 0.006 | - | - | - |
| *General comorbidities* | | | | | | |
| Cardiopathy | 11.7 | [2.89 – 59.5] | 0.001 | 10.22 | [1.42 – 102.6] | 0.03 |
| Diabetes | 3.49 | [0.91 – 13.06] | 0.06 |  |  |  |
| *Hepatic comorbidities* | | | | | | |
| Non cancer hepatopathy | 6.78 | [2.38 – 2.03] | < 0.001 | 4.55 | [1.19 – 18.51] | 0.03 |
| Cirrhosis | 4.72 | [1.58 – 14.35] | 0.005 | - | - | - |
| Portal hypertension | 4.06 | [0.88 – 18.82] | 0.06 |  |  |  |
| Steatosis | 4.06 | [0.88 – 18.82] | 0.06 |  |  |  |
| *Etiology of surgery* | | | | | | |
| Primitive CHC | 1 | - | - |  |  |  |
| Metastasis | 0.20 | [0.06 – 0.62] | 0.006 |  |  |  |
| Others | 0.26 | [0.05 – 1.07] | 0.08 |  |  |  |
| *Pre-surgery biological workup* | | | | | | |
| Abnormal GGT | 4.39 | [1.51 – 14.78] | 0.01 | 5.54 | [1.42 – 28.70] | 0.02 |
| Abnormal ASAT | 3.81 | [1.33 – 12.04] | 0.02 | - | - | - |
| Abnormal ALAT | 3.37 | [1.19 – 9.93] | 0.02 | - | - | - |
| Abnormal APL | 3.07 | [1.06 – 9.07] | 0.04 |  |  |  |
| *Surgery characteistics* | | | | | | |
| Ischemia duration in mn | 1.03 | [1.01 – 1.05] | 0.03 | 1.03 | [1.01 – 1.07] | 0.04 |
| *Aaesthetic characteristics* | | | | | | |
| Use of vasopressive drug(s) | 3.20 | [1.16 – 9.85] | 0.03 | 3.79 | [1.02 – 17.11] | 0.04 |
